# Radiomics analysis based on dynamic contrast-enhanced MRI for predicting early recurrence after hepatectomy in hepatocellular carcinoma patients

**DOI:** 10.1038/s41598-025-02291-6

**Published:** 2025-07-01

**Authors:** Kai-Di Wang, Ming-Jing Guan, Zi-Yang Bao, Zhe-Jin Shi, Hai-Hang Tong, Zun-Qiang Xiao, Lei Liang, Jun-Wei Liu, Guo-Liang Shen

**Affiliations:** 1 General Surgery, Cancer Center, Department of Hepatobiliary and Pancreatic Surgery and Minimal Invasive Surgery, Zhejiang Provincial People’s Hospital, Affiliated People’s Hospital, Hangzhou Medical College, Hangzhou, Zhejiang Province China; 2 Department of the Second School of Clinical Medicine, Zhejiang Chinese Medical University, Hangzhou, Zhejiang Province China

**Keywords:** Hepatocellular carcinoma, Radiomics, Machine learning, Early recurrence, Cancer imaging, Cancer models, Liver cancer

## Abstract

This study aimed to develop a machine learning model based on Magnetic Resonance Imaging (MRI) radiomics for predicting early recurrence after curative surgery in patients with hepatocellular carcinoma (HCC).A retrospective analysis was conducted on 200 patients with HCC who underwent curative hepatectomy. Patients were randomly allocated to training (*n* = 140) and validation (*n* = 60) cohorts. Preoperative arterial, portal venous, and delayed phase images were acquired. Tumor regions of interest (ROIs) were manually delineated, with an additional ROI obtained by expanding the tumor boundary by 5 mm. Radiomic features were extracted and selected using the Least Absolute Shrinkage and Selection Operator (LASSO). Multiple machine learning algorithms were employed to develop predictive models. Model performance was evaluated using receiver operating characteristic (ROC) curves, decision curve analysis, and calibration curves. The 20 most discriminative radiomic features were integrated with tumor size and satellite nodules for model development. In the validation cohort, the clinical-peritumoral radiomics model demonstrated superior predictive accuracy (AUC = 0.85, 95% CI: 0.74–0.95) compared to the clinical-intratumoral radiomics model (AUC = 0.82, 95% CI: 0.68–0.93) and the radiomics-only model (AUC = 0.82, 95% CI: 0.69–0.93). Furthermore, calibration curves and decision curve analyses indicated superior calibration ability and clinical benefit. The MRI-based peritumoral radiomics model demonstrates significant potential for predicting early recurrence of HCC.

**Supplementary Information:**

The online version contains supplementary material available at 10.1038/s41598-025-02291-6.

## Introduction

Primary liver cancer, predominantly manifested as hepatocellular carcinoma (HCC), represents one of the deadliest malignancies worldwide, constituting approximately 90% of liver malignancies and standing as the fourth most frequent cause of cancer mortality on a global scale^1,2^. For patients diagnosed with early-stage HCC, curative surgical resection remains the optimal therapeutic intervention, while ablative techniques serve as an alternative approach when operatively appropriate^3^. While patients with solitary tumors may achieve a 5-year survival rate of up to 70%, high recurrence rates continue to hinder efforts to improve survival outcomes. Post-operative tumor recurrence demonstrates a distinctive temporal pattern in HCC, with approximately 70% of recurrent cases manifesting within the initial 24-month period following surgical intervention^4^. This early recurrence substantially diminishes patients’ wellbeing and survival duration, markedly reducing post-operative survival time and acting as the primary factor affecting post-operative mortality^5–7^. Although various biomarkers and oncological characteristics associated with long-term HCC prognosis have been identified and have positively influenced diagnosis and treatment, their value in addressing post-operative recurrence and improving therapeutic efficacy remains limited and has not yielded the expected outcomes.

Although an increasing number of factors have been identified as related to early recurrence of HCC, traditional serological and pathological methods still have limitations in preoperative prediction of HCC recurrence^8^. Serological markers show poor predictive efficacy, while more valuable indicators can be obtained through pathological examination, which inevitably involves invasive procedures. Currently, imaging studies, particularly enhanced Magnetic Resonance Imaging (MRI), have become routine preoperative examinations for HCC, providing easily accessible and voluminous data. MRI accurately distinguishes tumors from liver tissue with superior soft tissue resolution, outperforming CT and ultrasound in detecting small lesions and analyzing hemodynamic changes, thus emerging as an optimal platform for radiomics research^9,10^. Extensive research has demonstrated that radiomics holds remarkable promise across diverse cancer-related applications. For example, researchers have employed MRI radiomics combined with machine learning to predict microvascular invasion in HCC, with the combination of clinical factors and radiomics models producing a nomogram with optimal diagnostic performance (AUC = 0.948)^11^. Contemporary research has established that the radiological characteristics within peritumoral regions serve as valuable imaging biomarkers for analytical investigation^12,13^,thereby improving predictive capabilities. However, the generalizability of these findings requires additional verification, with persistent challenges including restricted sample sizes and absence of independent cohort testing.

Advanced machine learning revolutionizes precision oncology by transforming personalized cancer treatment. Unlike conventional methods, ML integrates multi-dimensional data to build precise predictive models. AI and big data analytics reshape HCC clinical management, serving as vital decision-support tools for personalized therapies. This AI-enhanced approach improves prognoses while preserving clinical interpretability through dynamic risk stratification^14–16^. Empirical evidence from prior investigations has conclusively established the clinical value of integrating radiomics features with machine learning approaches for HCC recurrence prediction^17–19^. Despite the significant potential of MRI, current investigations exploring intratumoral and peritumoral radiomic signatures for hepatocellular carcinoma recurrence prediction remain in their nascent stages. This study innovatively integrates intratumoral and peritumoral features to develop a comprehensive tumor microenvironment predictive model, enabling precise stratification of early recurrence risk in HCC patients.

## Materials and methods

This study was approved by the Ethics Committee of Zhejiang Provincial People’s Hospital (Approval No. QT2023141) and conducted in accordance with the Declaration of Helsinki. All methods adhered to relevant guidelines and regulations. As this is a retrospective study, informed consent was waived by the Ethics Committee due to the anonymized use of clinical data.

### Patient population

**Fig. 1:**
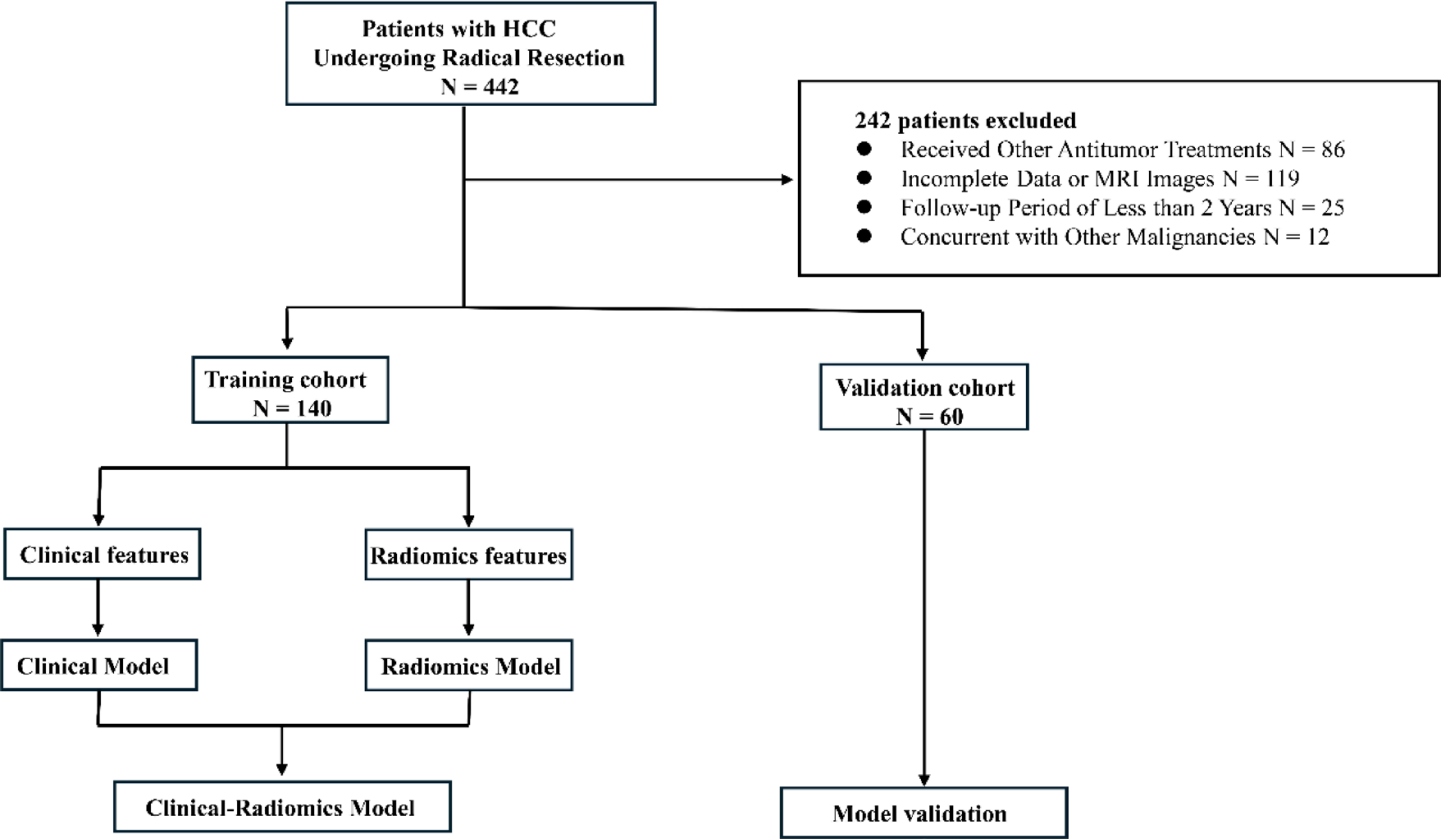
The flowchart of this study.

This single-center retrospective analysis encompassed a cohort of patients diagnosed with hepatocellular carcinoma who underwent radical hepatectomy at the Department of Hepatobiliary Surgery, Zhejiang Provincial People’s Hospital during a five-year period spanning from January 2017 through December 2021. The inclusion criteria included: (1) pathologically confirmed HCC, (2) underwent curative hepatic resection, and (3) received contrast-enhanced MRI examination within one month prior to surgery. Exclusion criteria consisted of: (1) previous HCC-related treatments including TACE, radiofrequency ablation, chemotherapy, or radiotherapy, (2) previous hepatic resection, (3) secondary liver cancer, (4) incomplete clinical data or suboptimal/missing imaging quality, and (5) follow-up period shorter than two years. In this research, the total sample (200 patients) underwent randomization, with 70% (140 patients) assigned to the training group and the remaining 30% (60 patients) comprising the validation cohort. Finally, clinical and imaging data from 140 patients were collected. Clinical variables identified through correlation analysis were combined with radiomics features extracted and selected from imaging data to establish machine learning models. A validation cohort was utilized to evaluate model performance. Figure 1 provides a visual representation of the overall study process and methodology employed.

### Clinicopathological characteristics

Potential influence on HCC recurrence were collected, including: age, gender, Body Mass Index(BMI), liver cirrhosis, alpha-fetoprotein (AFP) levels, carcinoembryonic antigen (CEA) levels, international normalized ratio (INR), hepatitis B infection status, alanine aminotransferase (ALT), aspartate aminotransferase (AST), gamma-glutamyl transferase (GGT), total bilirubin, albumin (ALB), Child-Pugh classification, performance status (PS) score, microvascular invasion (MVI), tumor number, maximum tumor diameter, tumor differentiation grade, satellite nodules, and surgical approach. BMI was determined by dividing patients’ weight in kilograms by the square of their height in meters. MVI was defined as the presence of tumor cells within the vascular space lined by endothelium, as identified by pathological examination^20^.

### Follow-up strategy

The post-operative follow-up protocol included laboratory tests and imaging examinations (e.g., CT, MRI, and ultrasound). During the initial two years post-surgery, patients were monitored every three months, followed by biannual check-ups thereafter. The study’s main outcome measure was early relapse, characterized as the emergence of novel intrahepatic tumors or distant metastases within 24 months after undergoing curative liver resection surgery^21^.Recurrence was primarily identified through imaging examinations and serum tumor biomarkers, and follow-up continued until December 25, 2023.

### MRI imaging protocol

MRI examinations were conducted no more than one month before the surgery, employing a 3.0T scanner (GE Healthcare, Milwaukee, WI, USA). The scanning parameters were as follows: repetition time (TR) 5.0 ms, echo time (TE) 1.1–2.9 ms, flip angle 30°, slice thickness 6–8 mm, image matrix 512 × 512, and pixel spacing 0.5–0.8 mm. Dynamic contrast-enhanced (DCE) imaging was performed using the conventional MR contrast agent gadolinium-diethylenetriamine pentaacetic acid (Gd-DTPA), administered at a dose of 0.2 mmol/kg body weight with an injection rate of 2.5 mL/s. The arterial phase scanning commenced approximately 15 s after injection, the portal phase at 50–70 s, and the equilibrium phase at 90–120 s. To minimize magnetic field inhomogeneities, image preprocessing included N4 bias field correction.

### Image analysis

**Fig. 2:**
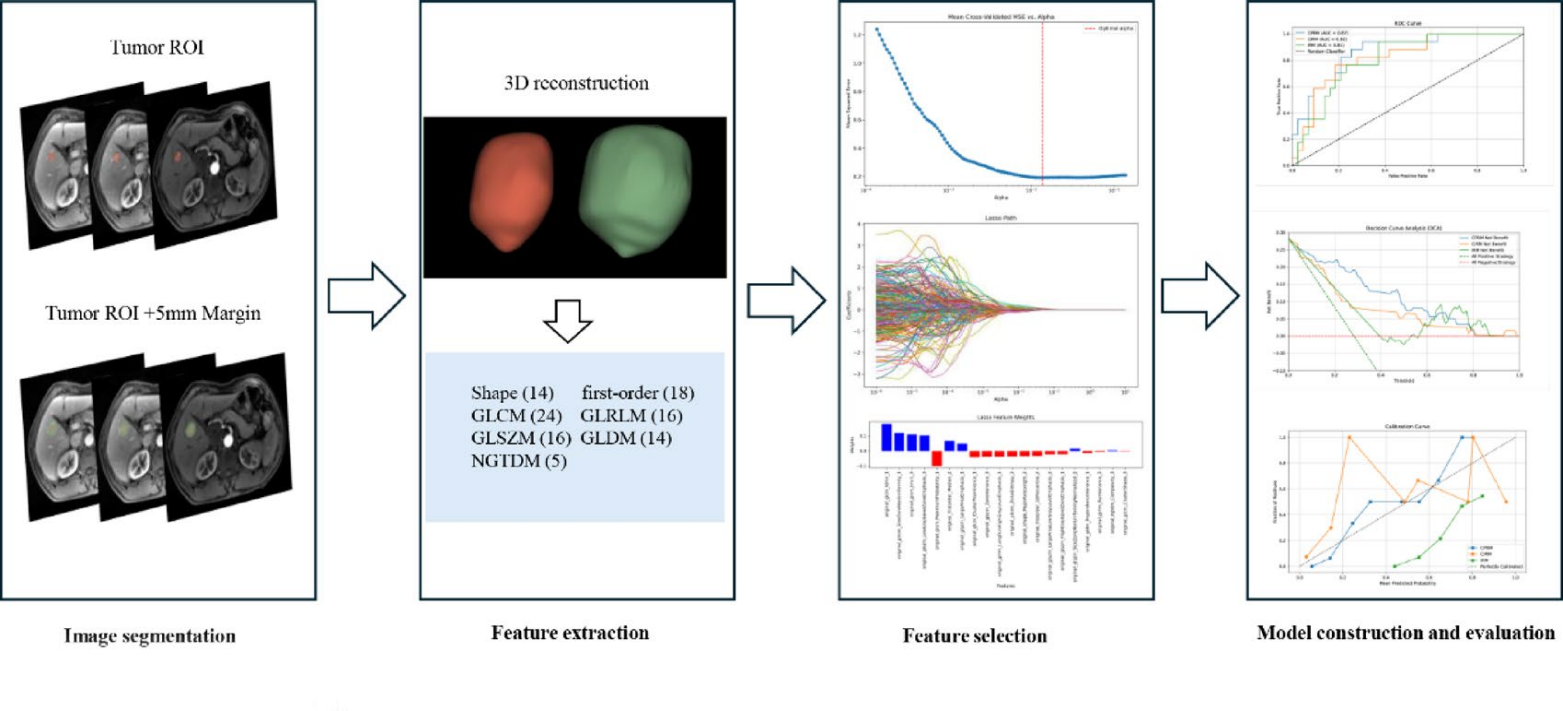
The flowchart of radiomics analysis.

The arterial, portal venous, and delayed phase MRI images underwent evaluation by two hepatobiliary surgeons who were blind to the clinical data. Three-dimensional (3D) regions of interest (ROIs) were manually segmented on a slice-by-slice basis using 3D-Slicer software (version 5.7.0). Subsequently, an automatic expansion band with a width of 5 mm was generated to facilitate the extraction of radiomic features from the peritumoral region. Image preprocessing was performed using the SimpleITK package in Python, and radiomic features were extracted from arterial, portal venous, and delayed phases using the open-source Python package PyRadiomics. All MRI datasets were resampled to achieve an isotropic voxel size of 1.0 mm³, thereby minimizing inconsistencies between different images. To assess the reliability of radiomic features, intra- and inter-observer consistency analyses were performed using the Pingouin library in Python on images from 30 randomly selected patients. Intra-observer correlation analysis was based on radiomic features extracted twice within a one-week interval by reader 1, while inter-observer correlation analysis was based on features extracted independently by readers 1 and 2. Radiomic features demonstrating intra- and inter-observer intraclass correlation coefficients (ICCs) exceeding 0.75 were chosen for further analysis (Supplementary Table 1). Prior to feature selection, Z-score normalization was employed to standardize the data. A total of 107 shape-, intensity-, and texture-based features were extracted from each three-dimensional ROI, with 321 features extracted for each lesion (intratumoral and peritumoral regions in arterial, portal venous, and delayed phases). The least absolute shrinkage and selection operator (LASSO) algorithm was employed to identify the most relevant features for predicting HCC recurrence in further analyses (Supplementary Fig. 1). Ultimately, two sets of ROIs were delineated and extracted: the tumor and the tumor boundary expanded by 5 mm. Each set incorporated features from three phase images. Following feature selection, these two sets of radiomics features were used to establish machine learning models, either independently or in combination with clinical variables. The flowchart of radiomics analysis is presented in Fig. 2.

### Model construction

Patients in the training set were used for model construction and algorithm development, while those in the validation set were used to assess the models’ predictive performance. Utilizing the Scikit-learn package in Python, we developed machine learning radiomic models encompassing logistic regression, support vector machine, decision tree, k-nearest neighbors, random forest, extreme gradient boosting (XGBoost), and categorical boosting (CatBoost) algorithms. Three models were constructed: the intratumoral radiomics model (IRM), the clinical-intratumoral radiomics model (CIRM) combining clinicopathological factors and intratumoral imaging features, and the clinical-peritumoral radiomics model (CPRM) integrating clinicopathological factors with both intratumoral and peritumoral imaging features.

### Statistical analysis

All statistical analyses were conducted using Python (version 3.9.2) in conjunction with relevant statistical packages, such as NumPy, SciPy, and scikit-learn. Categorical variables at baseline were compared between the recurrence and non-recurrence groups using either the chi-square test or Fisher’s exact test. The results were presented as frequencies (percentages). Continuous variables were compared between groups using the independent samples t-test or the Mann-Whitney U test, based on the normality of the data distribution. Results were expressed as either mean ± standard deviation (SD) or median (interquartile range [IQR]), corresponding to the distribution properties of the data. Univariate and multivariate logistic regression analyses were performed on clinical variables to identify potential risk factors associated with HCC recurrence. Variables that achieved a p-value < 0.05 in the univariate analysis were subsequently included in the multivariate logistic regression model for further evaluation.

## Result

### Patient characteristics

The study retrospectively reviewed 442 patients who underwent surgical treatment for HCC at Zhejiang Provincial People’s Hospital. The final study cohort consisted of 200 patients (160 males and 40 females) with a mean age of 58.3 ± 10.9 years. This cohort was further divided into a training set of 140 patients and a validation set of 60 patients for subsequent analysis. Early recurrence (ER) occurred in 66 patients, of whom 54 (81.8%) developed intrahepatic recurrence, 4 (6.7%) experienced extrahepatic recurrence, and 8 (12.1%) presented with both intrahepatic and extrahepatic recurrence. Table 1 summarizes the baseline characteristics of the training cohort and the validation cohort. The training cohort and the validation cohort showed no significant differences in the majority of variables, with the only significant difference observed in AFP (*P* = 0.045).

**Table 1:** Baseline characteristics of HCC patients.

	No. (%)			
Variables	Total (N = 200)	Training (N = 140)	Validation (N = 60)	*P* value
Age, year, mean ± SD	58.3 ± 10.9	58.6 ± 10.7	57.9 ± 11.3	0.704
Sex, n (%)				1.000
Male	160(80.0%)	114(81.4%)	46(76.7%)	
Female	40(20.0%)	26(18.6%)	14(23.3%)	
BMI, kg/m2, mean ± SD	23.4 ± 3.4	23.2 ± 3.4	23.7 ± 3.3	0.284
PS score, n (%)				0.709
0	163(81.5%)	115(82.1%)	48(80.0%)	
1–2	37(18.5%)	25(17.9%)	12(20.0%)	
Liver cirrhosis, n (%)				0.732
No	47(23.5%)	38(27.1%)	9(15.0%)	
Yes	153(76.5%)	102(72.9%)	51(85.0%)	
HBV, n (%)				0.396
No	35(17.5%)	25(17.9%)	10(16.7%)	
Yes	165(82.5%)	115(82.1%)	50(83.3%)	
Child–pugh, n (%)				1.000
A	191(95.5%)	135(96.4%)	56(93.3%)	
B	9(4.5%)	5(4.6%)	4(6.7%)	
Tumor size, mm, median (IQR)	35.0 (22.0—50.0)	35.0 (21.8—45.0)	33.5 (22.0—60.0)	0.344
Differentiation, n (%)				0.839
Moderate-high	149(74.5%)	110(79.6%)	39(65.0%)	
Low-undifferentiated	51(25.5%)	30(21.4%)	21(35.0%)	
Tumor capsule, n(%)				0.304
Intact	48(24.0%)	36(25.7%)	12(20.0%)	
Disrupted/absent	152(76.0%)	104(74.3%)	48(80.0%)	
Satellite lesions, n(%)				1.000
No	188(94.0%)	131(93.6%)	57(95.0%)	
Yes	12(6.0%)	9(6.4%)	3(5.0%)	
Tumor number, n(%)				1.000
Solitary	171 (85.5%)	123(87.9%)	48(80.0%)	
Multiple	29 (14.5%)	17(12.1%)	12(20.0%)	
MVI, n(%)	92 (46.0%)	57(40.7%)	35(58.3%)	0.236
Surgical approach, n (%)				0.789
Laparoscopy	186(93.0%)	132(94.3%)	54(90.0%)	
Laparotomy	14 (7.0%)	8(5.7%)	6(10.0%)	
Hospital stays, day, median (IQR)	8.0 (6.0—10.0)	8.0 (6.0—10.0)	8.5 (7.0—11.3)	0.113
AFP, ng/ml, median (IQR)	29.6 (4.7—368.1)	21.1 (4.0—253.1)	90.8 (6.3—747.6)	0.045
CEA, ng/ml, median (IQR)	2.4 (1.0—3.5)	2.2 (1.0—3.4)	2.6 (1.4—3.6)	0.501
CA19-9, U/ml, median (IQR)	15.0 (9.8—29.7)	15.4 (9.4—29.7)	14.4 (11.1—28.0)	0.582
INR, median (IQR)	1.0 (1.0—1.1)	1.0 (1.0—1.1)	1.0 (1.0—1.1)	0.399
ALT, U/L, median (IQR)	28.0 (19.0—44.0)	28.5 (20.0—43.0)	28.0 (18.0—55.3)	0.901
AST, U/L, median (IQR)	33.0 (25.0—44.0)	33.0 (26.0—43.3)	36.5 (24.0—49.3)	0.582
GGT, U/L, median (IQR)	48.0 (29.0—91.0)	44.0 (29.0—85.0)	57.0 (29.5—92.3)	0.290
TBIL, μmol/L, median (IQR)	15.0 (10.8—19.7)	15.4 (10.9—20.7)	40.3 (36.9—43.3)	0.380
ALB, g/L, median (IQR)	39.5 (36.0—42.5)	39.3 (36.0—42.2)	39.7 (37.1—42.9)	0.437

*SD* standard deviation, *IQR* Interquartile range, *BMI* Body mass index, *PS* Performance status, *HBV* Hepatitis B virus, *MVI* Microvascular invasion, *AFP* Alpha fetoprotein, *CEA* Carcinoembryonic antigen, *CA19-9* carbohydrate antigen 19–9, *INR* International normalized ratio, *ALT* Alanine aminotransferase, *AST* Aspartate aminotransferase, *GGT* Gamma-glutamyl transferase, *TBIL* Total bilirubin, *ALB* Albumin.

### Radiomic feature extraction

A total of 321 radiomic features were extracted for each patient, consisting of 107 features from each of the three phases of the imaging series: arterial, portal venous, and delayed. The radiomic features extracted included 14 shape-based features, 18 first-order statistical features, and 75 texture-based features. Among the texture-based features, 24 were derived from the Gray Level Co-occurrence Matrix (GLCM), 16 from the Gray Level Run Length Matrix (GLRLM), 16 from the Gray Level Size Zone Matrix (GLSZM), 14 from the Gray Level Dependence Matrix (GLDM), and 5 from the Neighborhood Gray-Tone Difference Matrix (NGTDM). Following the intra- and inter-observer consistency analysis, features with intra- and inter-observer intraclass correlation coefficients (ICCs) greater than 0.75 were retained. After excluding 55 redundant features, a total of 266 features were retained for further analysis. The LASSO regression algorithm was applied to select the top 20 most important features.

### Clinical factor analysis

Univariate and multivariate logistic regression analyses were conducted on the included clinicopathological variables to identify potential risk factors associated with early HCC recurrence, with the results detailed in Table 2. In the univariate analysis, albumin (ALB) levels (*P* = 0.031), CA19-9 levels (*P* = 0.006), tumor size (*P* = 0.001), PS score (*P* = 0.008), and presence of satellite lesions (*P* = 0.010) were identified as factors associated with early HCC recurrence. Variables demonstrating a P-value less than 0.05 in the univariate analysis were subsequently incorporated into the multivariate logistic regression model. The multivariate analysis identified tumor size (*P* = 0.036) and presence of satellite lesions (*P* = 0.011) as independent risk factors for early HCC recurrence.

**Table 2:** Univariate and multivariable logistic regression analysis of clinical factors associated with early recurrence in HCC patients.

	Univariate logistic analysis		Multivariate logistic analysis	
Variables	*P* value	OR (95%CI)	*P* value	OR (95%CI)
Age	0.888	1.026 (0.712–1.469)		
Sex	0.258	1.223 (0.862–1.735)		
BMI	0.409	0.860 (0.600–1.231)		
Liver cirrhosis	0.074	1.404 (0.968–2.037)		
CA-199	0.006	1.789 (1.178–2.718)	0.150	1.420 (0.880–2.292)
AFP	0.598	0.547 (0.305–7.834)		
CEA	0.335	0.837 (0.584–1.201)		
HBV	0.569	1.111 (0.773–1.597)		
ALT	0.290	1.260 (0.821–1.932)		
AST	0.092	1.379 (0.949–2.005)		
GGT	0.106	1.325 (0.942–1.864)		
TBIL	0.151	0.747 (0.502–1.119)		
INR	0.680	1.075 (0.764–1.512)		
ALB	0.031	0.662 (0.455–0.964)	0.356	0.818 (0.534–1.253)
Child–pugh	0.541	0.866 (0.547–1.372)		
Tumor size	0.001	2.067 (1.323–3.230)	0.036	1.725 (1.036–2.873)
Differentiation	0.349	1.190 (0.827–1.712)		
Tumor capsule	0.944	1.012 (0.718–1.428)		
Tumor number	0.438	1.156 (0.802–1.665)		
Surgical approach	0.774	0.946 (0.649–1.379)		
PS score	0.008	1.590 (1.126–2.245)	0.076	1.427 (0.963–2.114)
Hospital stays	0.163	1.299 (0.900–1.875)		
Surgical time	0.056	1.413 (0.991–2.014)		
Satellite Lesions	0.010	1.651 (1.125–2.423)	0.011	1.711 (1.131–2.588)

*BMI* Body mass index, *PS* Performance status, *HBV* Hepatitis B virus, *AFP* Alpha fetoprotein, *CEA* Carcinoembryonic antigen, *CA19-9* carbohydrate antigen 19–9, *INR* International normalized ratio, *ALT* Alanine aminotransferase, *AST* Aspartate aminotransferase, *GGT* Gamma-glutamyl transferase, *TBIL* Total bilirubin, *ALB* Albumin.

### MR radiomic analysis and model comparison

**Fig. 3:**
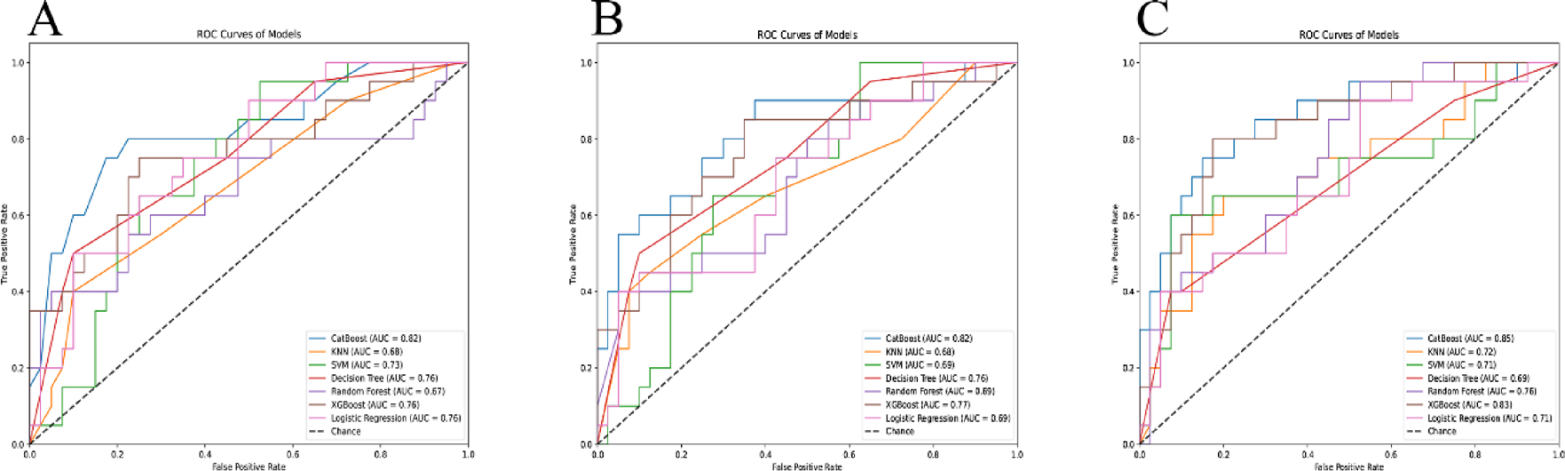
ROC curves for the Intratumoral Radiomics Model (IRM), Clinical Intratumoral Radiomics Model (CIRM), and Clinical Peritumoral Radiomics Model (CPRM). (**A**) Receiver Operating Characteristic (ROC) Curves of Different Machine Learning Models in Intratumoral Radiomics Model, (**B**) Receiver Operating Characteristic (ROC) Curves of Different Machine Learning Models in Clinical Intratumoral Radiomics Model, (**C**) Receiver Operating Characteristic (ROC) Curves of Different Machine Learning Models in Clinical Peritumoral Radiomics Model. *ROC* Receiver Operating Characteristic curves, *AUC* Area Under the Receiver Operating Characteristic Curve, *KNN* K-Nearest Neighbors, *SVM* Support Vector Machine, *XGBoost* eXtreme Gradient Boosting, *CatBoost* Categorical Boosting.

**Fig. 4:**
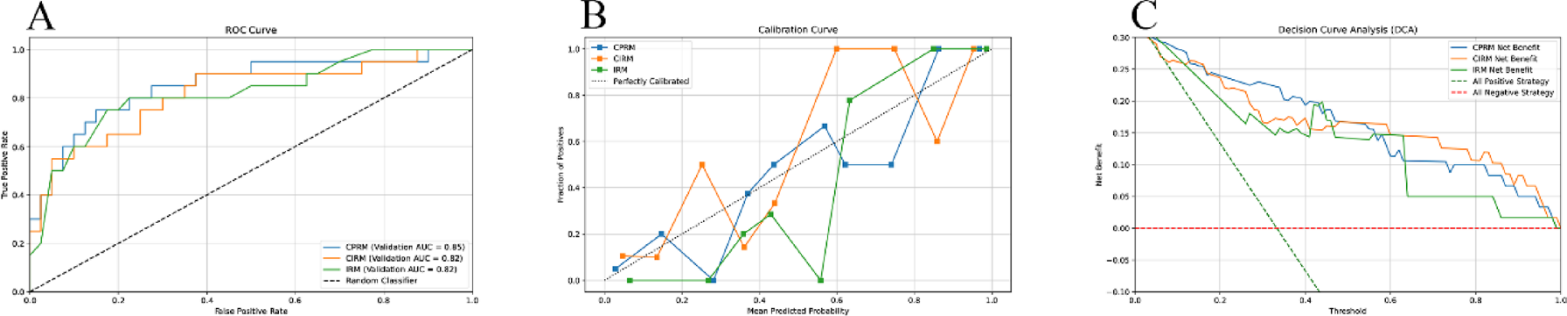
Predictive performance of the imaging radiomics model (IRM), clinical-intratumoral radiomics model (CIRM), and clinical-peritumoral radiomics model (CPRM) in predicting early recurrence of HCC. (**A**) ROC curves; (**B**) Calibration curves; (**C**) Decision curve analysis.

Seven machine learning algorithms were employed to construct the intratumoral radiomics model (IRM), with the categorical boosting (CatBoost) algorithm demonstrating the best performance, achieving an AUC of 0.82 in the validation set and an AUC of 0.76 in the training set. Similarly, seven machine learning algorithms were used to construct the clinical-intratumoral radiomics model (CIRM) and the clinical-peritumoral radiomics model (CPRM), with the CatBoost algorithm showing the best performance in both models, achieving AUCs of 0.82 and 0.85 in the validation set, and AUCs of 0.98 and 0.94 in the training set, respectively. The comparative performance of the three models across multiple machine learning algorithms is presented in Table 3. The receiver operating characteristic (ROC) curves of various machine learning models are depicted in Fig. 3. DCA curves and Calibration curves were plotted for all three models to assess their calibration and clinical utility, respectively(results shown in Fig. 4). In the CatBoost algorithm, the CPRM demonstrated the highest AUC value (0.852) and sensitivity (60%), indicating its slight superiority in identifying recurrence compared to the other two models. The CIRM exhibited the highest specificity (95%) and accuracy (81.70%). The IRM performed comparably or worse than the other two models across all metrics. The calibration curves were assessed using the Hosmer-Lemeshow Test, which showed p-values greater than 0.05 for all three models (0.1419, 0.1206, and 0.2800), demonstrating good calibration. The Brier scores were calculated, with CPRM achieving the lowest score (0.1373), followed by CIRM (0.1503), and IRM with the highest (0.1780), further confirming that CPRM had the highest prediction accuracy. Performance metrics included NRI and IDI values, with both CPRM and CIRM showing positive NRI (0.0750) and IDI (approximately 0.20) compared to IRM. Nevertheless, the DeLong Test results revealed that p-values between all model pairs were greater than 0.05, indicating that the differences in ROC curves were not statistically significant.

**Table 3:** Comparative performance of models across multiple machine learning algorithms.

	Model	AUC (95% CI)	Sensitivity (%)	Specificity (%)	Accuracy (%)
CatBoost	IRM	0.821 (0.688–0.931)	50.00	92.50	78.30
	CIRM	0.818 (0.683–0.930)	55.00	95.00%	81.70
	CPRM	0.852 (0.728–0.944)	60.00	90.00	80.00
KNN	IRM	0.680 (0.533–0.820)	40.00	90.00	73.30
	CIRM	0.679 (0.531–0.819)	45.00	87.50	73.30
	CPRM	0.693 (0.544–0.839)	45.00	80.00	68.30
SVM	IRM	0.726 (0.595–0.854)	40.00	82.50	68.30
	CIRM	0.559 (0.399–0.711)	0.00	100.00	66.70
	CPRM	0.703 (0.530–0.850)	10.00	97.50	68.30
Decision tree	IRM	0.756 (0.634–0.874)	50.00	90.00	76.70
	CIRM	0.756 (0.634–0.874)	50.00	90.00	76.70
	CPRM	0.685 (0.548–0.814)	40.00	90.00	73.30
Random forest	IRM	0.670 (0.508–0.830)	40.00	92.50	75.00
	CIRM	0.689 (0.542–0.832)	40.00	92.50	75.00
	CPRM	0.755 (0.625–0.874)	40.00	92.50	75.00
XGBoost	IRM	0.759 (0.612–0.893)	95.00	17.50	43.30
	CIRM	0.771 (0.632–0.897)	100.00	5.00	36.70
	CPRM	0.829 (0.705–0.928)	95.00	32.50	53.30
Logistic regression	IRM	0.759 (0.629–0.886)	0.00	100.00	66.70
	CIRM	0.691 (0.547–0.834)	40.00	90.00	73.30
	CPRM	0.711 (0.559–0.848)	40.00	90.00	73.30

*KNN* K-nearest neighbors, *SVM* Support vector machine, *XGBoost* eXtreme gradient boosting, *CatBoost* Categorical boosting, *IRM* IntratumoralImaging radiomics model, *CIRM* Clinical intratumoral radiomics model, *CPRM* Clinical peritumoral radiomics model.

## Discussion

In this study, we developed an MRI-based radiomics approach that demonstrated strong predictive performance for early recurrence of HCC. Our clinical-peritumoral radiomics model, which integrated clinical risk factors with peritumoral characteristics, achieved the best performance in the validation cohort with an AUC of 0.85 (95% CI, 0.72–0.95). This model outperformed those relying solely on intratumoral features or clinical variables, suggesting enhanced capability for predicting early recurrence of HCC. The CPRM also showed better predictive calibration with a lower Brier score (0.1373 vs. 0.1780) compared to the IRM. Among the top 20 features ranked by importance in the CPRM, 7 were from the Arterial Phase, 5 from the Portal Venous Phase, and 8 from the Delayed Phase. These features were predominantly texture-based, such as GLCM and GLSZM features^22^, accounting for 90% of the top 20 features. These texture features primarily describe the heterogeneity of gray-level intensity distribution within the tumor and characterize spatial relationships between pixels, thereby reflecting tumor heterogeneity. The quantification of tumor heterogeneity through these radiomic features may facilitate personalized treatment planning and decision-making for HCC patients^23^.

Our clinical analysis identified several significant risk factors for early recurrence, including tumor size and the presence of satellite lesions, which were confirmed as independent risk factors. Additional factors associated with early recurrence included albumin levels, CA19-9 levels, and PS score^24,25^. These factors have also been established as predictors of HCC recurrence in previous studies^26–29^. We observed that the CIRM showed improved predictive performance compared to the IRM alone, with further improvement achieved by incorporating peritumoral radiomic features. In our machine learning approach, we observed superior performance with ensemble learning algorithms, particularly the CatBoost algorithm. This may be attributed to our study’s incorporation of multiple heterogeneous data sources, including clinicopathological, intratumoral, and peritumoral data, which have been shown to be effectively handled by CatBoost^30^.

Our findings extend previous research in several important ways. Prior studies such as Quan et al. analyzed radiomic features extracted from the largest cross-sectional area of HCC lesions on CT images, utilizing a peritumoral region extending 2 cm from the lesion boundary, achieving an AUC of 0.79 (95% CI, 0.66–0.92). However, their study extracted features only from a single cross-sectional slice, neglecting three-dimensional (3D) tumor information^31^. In contrast, our study explored a peritumoral region extending 5 mm from the entire 3D tumor volume, potentially capturing more comprehensive tumor information compared to previous 2D cross-sectional approaches. HCC recurrence is closely associated with MVI^32^. Previous studies have shown that using radiomics features from a 5 mm peritumoral region can effectively predict the occurrence of MVI^33,34^, potentially capturing sufficient peritumoral information while reducing interference from adjacent large blood vessels or bile ducts.

Peritumoral radiomic features in our model showed higher importance compared to intratumoral features, suggesting they may better capture the characteristics of the tumor microenvironment and heterogeneity. This aligns with previous studies showing that the tumor microenvironment in peritumoral liver tissue differs significantly from that within the tumor and is considered prognostically relevant, characterized by factors such as CD68 + and CD206 + macrophage densities^35^.

The identification of tumor size and satellite nodules as independent risk factors for early recurrence is consistent with previous studies. Similarly, our finding that incorporating clinical factors improves model performance aligns with existing literature. A study by Kim et al. observed optimal prognostic performance with a 3 mm peritumoral boundary extension, which is consistent with our results using a 5 mm extension, although the optimal extent of the peritumoral region requires further investigation^36^.

Early recurrence of HCC remains a critical challenge that adversely affects patient prognosis and treatment outcomes. Given the high recurrence rates, accurate early prediction of recurrence risk has the potential to significantly improve individualized patient management strategies. Our radiomics approach facilitates personalized diagnostic and treatment planning by quantifying tumor heterogeneity through radiomic features. From a clinical application perspective, even modest improvements in predictive performance may be meaningful in certain high-risk populations or specific clinical scenarios. For instance, in settings requiring high-sensitivity screening, the increased sensitivity of CPRM could have substantial impact on patient management.

However, our study has several limitations that should be acknowledged. First, as a single-center retrospective study with a relatively small sample size, our findings may be subject to potential selection bias or limited statistical power. Second, our results may have limited generalizability, as the enrolled patients were exclusively Asian. This may not fully reflect the characteristics of early HCC recurrence across different geographic regions, ethnic backgrounds, and socioeconomic levels^37^.Therefore, to ensure the generalizability and external validity of our findings, further validation through multi-center studies with larger and more diverse cohorts is necessary. Furthermore, although our study suggested potential biological mechanisms of HCC recurrence through radiomic features, the underlying molecular pathways remain unclear, despite the potential biological correlations. Further validation using molecular biology techniques is required to elucidate these mechanisms. Future studies should aim to integrate radiomic features with multi-omics data to better understand the biological significance of these features at the molecular level. Future research should validate the model through multicenter prospective trials, integrate postoperative histopathological data to establish dynamic optimization systems, decode radiomic biomarker mechanisms via genomic-transcriptomic-proteomic multi-omics integration, and extend this framework to other solid tumors, ultimately advancing radiomics-based personalized therapeutic decision-making systems.

## Conclusion

In conclusion, our study demonstrates the predictive value of MRI-based radiomics for early recurrence of HCC. Our machine learning models incorporating peritumoral radiomic features displayed enhanced predictive performance compared to those based solely on intratumoral or clinicopathological features. This radiomics approach offers significant clinical utility as a non-invasive tool for pre-treatment risk stratification, enabling clinicians to identify high-risk patients who may benefit from more aggressive interventions or intensified surveillance. As MRI is routinely performed in HCC management, this method could be readily integrated into clinical workflows to guide personalized treatment decisions, potentially improving patient outcomes without additional testing burden.

## Electronic supplementary material

Below is the link to the electronic supplementary material.

Supplementary Material 1

Supplementary Material 2

Supplementary Material 3

## Data Availability

The datasets used and analyzed during the current study are available from the corresponding author upon reasonable request.
