# Supplementary figures and images for: Radiomics analysis based on dynamic contrast-enhanced MRI for predicting early recurrence after hepatectomy in hepatocellular carcinoma patients
Source: Sci Rep. 2025 Jul 1;15:22240. doi: 10.1038/s41598-025-02291-6 (PMC12215142; doi:10.1038/s41598-025-02291-6)

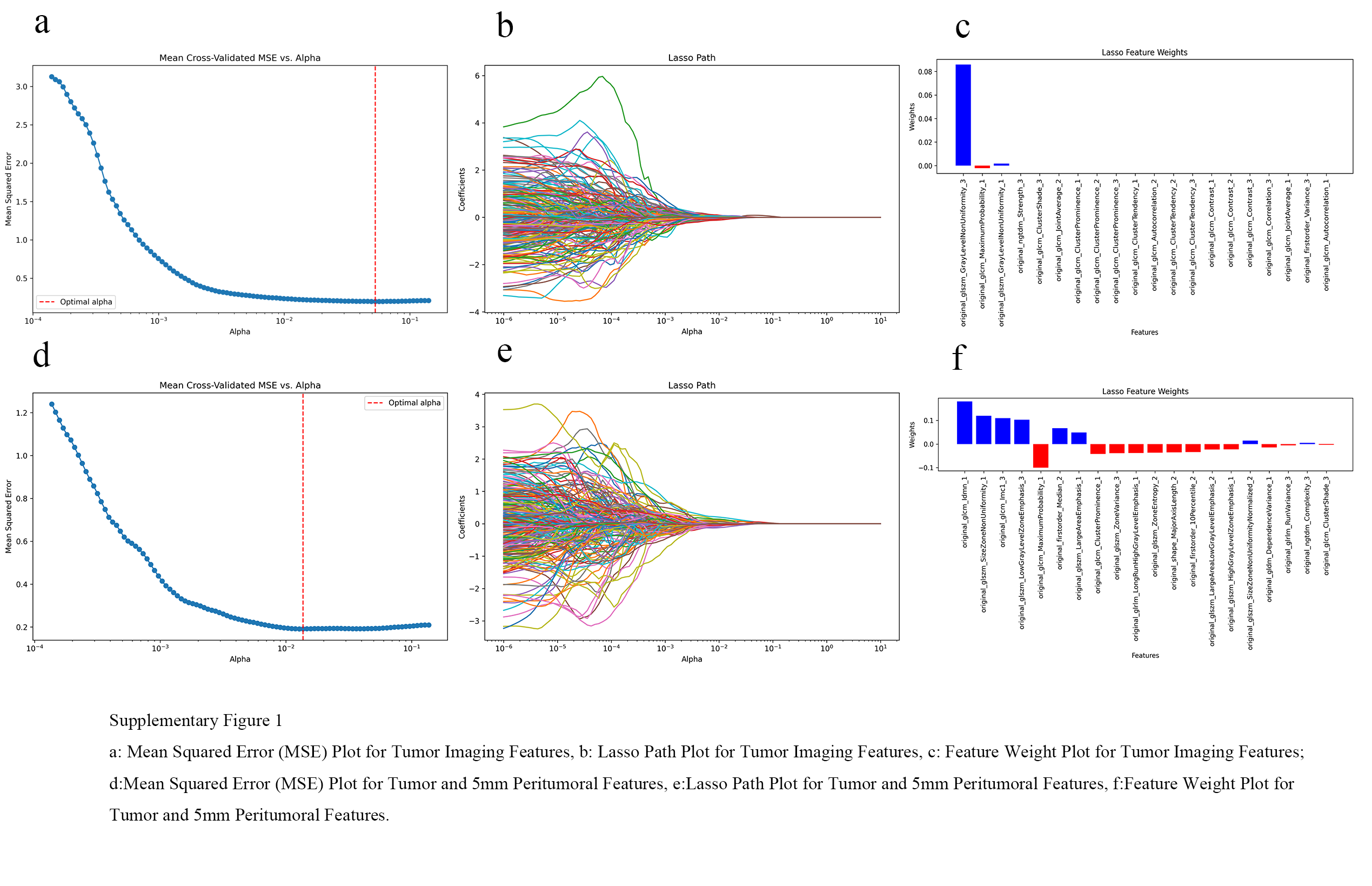

Supplement: Supplementary file 2 — Supplementary Material 2 [file 41598_2025_2291_MOESM2_ESM.png]

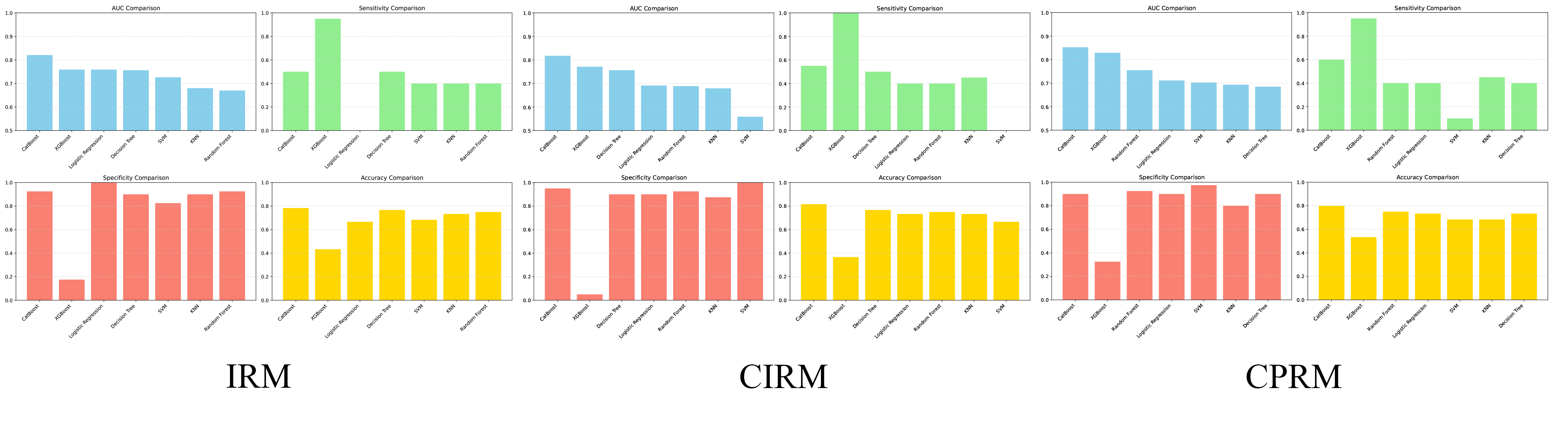

Supplement: Supplementary file 3 — Supplementary Material 3 [file 41598_2025_2291_MOESM3_ESM.tif]
